# Thromboembolic and Bleeding Events in Transthyretin Amyloidosis and Coagulation System Abnormalities: A Review

**DOI:** 10.3390/jcm12206640

**Published:** 2023-10-20

**Authors:** Angela Napolitano, Laura De Michieli, Giulio Sinigiani, Tamara Berno, Alberto Cipriani, Luca Spiezia

**Affiliations:** 1 General Internal Medicine & Thrombotic and Haemorrhagic Diseases Unit, Department of Medicine, Padova University Hospital, 35128 Padova, Italy; angela.napolitano@studenti.unipd.it; 2 Department of Cardiothoracic and Vascular Sciences & Public Health, Padova University Hospital, 35128 Padova, Italy; laura.demichieli@phd.unipd.it (L.D.M.); giulio.sinigiani@aopd.veneto.it (G.S.); alberto.cipriani@unipd.it (A.C.); 3 Haematology Unit, Department of Medicine, Padova University Hospital, 35128 Padova, Italy; tamara.berno@aopd.veneto.it

**Keywords:** thromboembolic events, bleeding events, transthyretin amyloidosis

## Abstract

Transthyretin amyloidosis (ATTR) is a group of diseases caused by the deposition of insoluble fibrils derived from misfolded transthyretin, which compromises the structure and function of various organs, including the heart. Thromboembolic events and increased bleeding risk are among the most important complications of ATTR, though the underlying mechanisms are not yet fully understood. Transthyretin plays a complex role in the coagulation cascade, contributing to the activation and regulation of the coagulation and fibrinolytic systems. The prevalence of atrial fibrillation, cardiac mechanical dysfunction, and atrial myopathy in patients with ATTR may contribute to thrombosis, though such events may also occur in patients with a normal sinus rhythm and rarely in properly anticoagulated patients. Haemorrhagic events are modest and mainly linked to perivascular amyloid deposits with consequent capillary fragility and coagulation anomalies, such as labile international-normalised ratio during anticoagulant therapy. Therefore, it is paramount to carefully stratify the thrombotic and haemorrhagic risks, especially when initiating anticoagulant therapy. Our review aims to ascertain the prevalence of thromboembolic and haemorrhagic events in ATTR and identify potential risk factors and predictors and their impact on antithrombotic therapy.

## 1. Introduction

Amyloidosis is a group of rare and underrecognised diseases characterised by the extracellular deposition of misfolded proteins that aggregate into insoluble amyloid fibrils [1]. Amyloid fibrils can deposit in multiple organs and tissues, thus altering their structure and function. Amyloidosis can be systemic or localised, inherited or acquired, and different forms have been characterised according to the type of precursor protein [2]. The three most frequent types of systemic amyloidosis that may involve the heart are: (i) monoclonal immunoglobulin light-chain (AL) amyloidosis; (ii) hereditary transthyretin (TTR) amyloidosis (ATTRv); and (iii) wild-type TTR amyloidosis (ATTRwt). AL amyloidosis is caused by a clonal plasma cell disorder, wherein the pathogenetic clone produces excess immunoglobulin light chains, resulting in amyloid formation and deposition [3]. This form is characterised by an aggressive clinical course and poor survival, if not diagnosed early [4]. ATTR amyloidosis stems from a misfolded TTR protein; ATTRwt amyloidosis is age-related and predominantly affects men > 70 years old, while the clinical manifestations of ATTRv are strongly related to the specific genetic variants [5]. In all these forms, cardiac involvement and the severity of cardiac amyloidosis (CA) are the main determinants of prognosis [6]. The cardinal symptom of patients with CA is heart failure with preserved ejection fraction (HFpEF) [7,8]. However, patients may also frequently encounter conduction disorders and arrhythmias [9,10]. Among the most clinically relevant complications of systemic amyloidosis are thrombotic and haemorrhagic events, particularly in CA patients [11]. The mechanisms underlying these events are only partially known, most notably with regard to ATTR amyloidosis. This narrative review aims to summarise the latest epidemiological data on thrombotic and bleeding events in ATTR amyloidosis and to identify their determinants and predictors and how they affect antithrombotic therapy.

## 2. Interactions between Transthyretin and the Coagulation System

**Figure 1:**
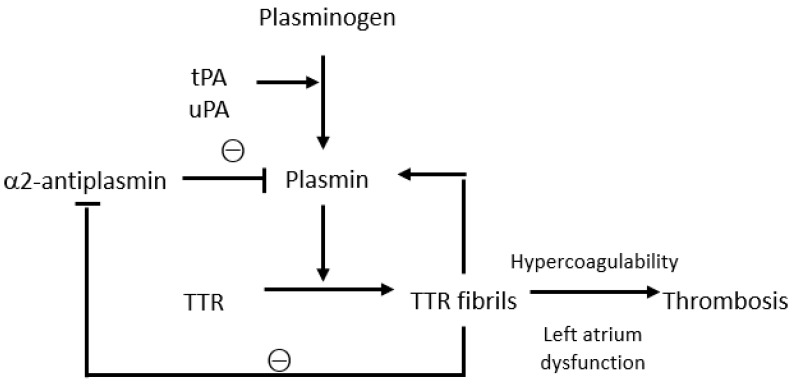
Interactions between the fibrinolytic system and transthyretin. tPA, tissue plasminogen activator; uPA, urokinase plasminogen activator; TTR, transthyretin.

Transthyretin is a tetrameric protein produced primarily by the liver, with smaller quantities being produced by the choroid plexus and retinal pigmented epithelial cells; it functions as a transporter protein for thyroxine and retinol-binding proteins [12]. ATTR amyloid also appears to play a role in the activation and regulation of the coagulation and fibrinolytic system (Figure 1). The pathogenesis of ATTR amyloidosis is a complex and not yet fully understood process, involving several mechanisms, and physiological fibrinolysis in vivo may be interconnected with amyloid formation. In fact, plasmin can cleave TTR, resulting in the formation of TTR fibrils containing truncated TTR molecules similar to the fibrils isolated from TTR amyloid formed in vivo. Notably, by measuring the levels of the plasmin–α2-antiplasmin complex, it has been demonstrated that a hyperfibrinolytic state is present mainly in patients with AA and AL amyloidosis, but not in those with TTR amyloidosis [13]. TTR plays a more complex role as amorphous extracellular protein aggregates bind to plasmin, thus contributing to plasmin activation and protecting plasmin from α2-antiplasmin inhibition. There is a self-contained regulatory system involving plasmin activation by amyloid and amyloid degradation by plasmin [14]. Circulating TTR can diffuse to the extracellular compartment and become trapped in or leak from the fibrin clot. Plasminogen is converted to plasmin by uPA in the extracellular compartment and by tPA in the clot. The binding of tPA and plasminogen to fibrin occurs in a lysine-dependent manner. Plasmin cleaves tetrameric TTR and releases a truncated residue 49–127 C-terminal fragment and full-length protomers that can generate fibrillar nuclei that aggregate into amyloid fibrils and accumulate in the extracellular space [12]. The fibrinolytic system appears to be linked to the onset of spontaneous intracerebral haemorrhage (ICH) in patients with cerebral amyloid angiopathy (CAA) who present a pathological amyloid beta (Aβ) deposition in the brain vasculature (i.e., cortical and leptomeningeal arteries, arterioles, and capillaries). Mutated Aβ in hereditary cerebral amyloid angiopathy enhances tPA-mediated plasminogen activation, alters clot structure, delays fibrinolysis, and contributes to the haemorrhagic and ischaemic manifestations of CAA. Normally, plasminogen activation degrades both Aβ and fibrin. However, in the presence of excessive plasmin generation due to elevated Aβ and vascular wall damage due to Aβ deposition and alterations of the cerebral microvasculature, plasminogen activation may increase the risk of spontaneous ICH due to hyperfibrinolysis, complicating r-tPA thrombolysis in acute ischaemic stroke [15]. In addition to the fibrinolytic system, TTR also plays a role in the coagulation system. Beta amyloid can bind to fibrinogen and cause fibrin clots to become structurally altered and resistant to fibrinolysis (protease-resistant amyloid–fibrin clot). X-ray crystallography revealed the binding of Abeta42 to the D-fragment of fibrinogen, which induces a structural change in the latter, thus blocking the cleavage of fibrin by plasmin [16]. Therefore, TTR has been found in platelet-rich fibrin and the formation of functional TTR amyloid in coagulation processes may contribute to both blood clotting and wound healing. Furthermore, the binding of amyloid to fibrin delays the proteolysis of amyloid by plasmin, creating a balance between the activation of plasmin by amyloid and the degradation of amyloid by plasmin [17]. TTR levels are also strongly associated with incident VTE, as shown in a case–control study that collected blood samples from 200 patients with ATTR before VTE events occurred. In a panel of 46 potential predictors of VTE, transthyretin was identified as the strongest biomarker with a nominal *p*-value of 0.00015, and there is a dire correlation between plasma TTR levels and procoagulant activity in the blood [18]. A few studies conducted in patients with Coronavirus disease 2019 (COVID-19) found that plasma TTR levels correlated directly with the hypercoagulable state responsible for thrombotic complications, with a simultaneous reduction in TTR degradation by plasmin [19]. Abnormalities of the coagulation and fibrinolytic systems also appear to contribute to haemorrhagic events, as demonstrated in patients with familial amyloid polyneuropathy (FAP) who show significantly lower levels of prothrombin fragment 1 + 2 compared to the controls, though the role of transthyretin remains unclear [20].

## 3. Thrombotic Events in Transthyretin Amyloidosis

Thrombotic events are among the most important complications of both AL and ATTR amyloidosis, especially with cardiac involvement [21,22]. However, studies that investigated the incidence, characteristics, and risk factors of thrombotic events in patients with ATTR amyloidosis are scarce and mostly include small single-centre cohorts (Table 1). Intracardiac thrombi and cerebral ischemic events have been described among major thromboembolic events in ATTR amyloidosis, whereas peripheral and gastrointestinal thrombosis are less common [21]. Patients with transthyretin amyloid cardiomyopathy (ATTR-CM) showed a high prevalence of atrial fibrillation (AF) (88%), which occurred more frequently in ATTR vs. AL. Amyloid infiltration of the atria and myocardium is thought to predispose patients to AF and high thromboembolic risk [23,24]. To date, whether a hypercoagulable state may contribute to the thrombotic diathesis and its underlying mechanisms are unknown. There have been reports noting the high prevalence of intracardiac thrombosis not only in patients with AF but also those with a normal sinus rhythm. A study by Vilches et al. [22] found an incidence rate of 0 per 100 patient-years among patients with a normal sinus rhythm undergoing oral anticoagulant treatment (OAT); 1.3 among those with a normal sinus rhythm without OAT; 1.7 among those with AF undergoing OAT; and 4.8 among those with AF without OAT. The amyloid infiltration of the left atrium may result in atrial mechanical dysfunction and endothelial damage with consequent blood flow stasis, in particular in the presence of atrial stiffness and the electromechanical dissociation of the left atrium [23,24]. Moreover, diuretic therapy in the presence of concomitant heart failure is also thought to affect Virchow’s triad, causing abnormalities in factors involved in clotting and contributing to clot formation even in subjects with a normal sinus rhythm [25]. Understanding the underlying mechanisms of thromboembolism, in particular whether the coagulation system is involved, is paramount to ascertain which patients would benefit from an early anticoagulation treatment. The latest European Society of Cardiology (ESC) consensus document on myocardial and pericardial diseases recommends anticoagulant therapy in all patients with AF regardless of the CHA2SD2-VASc score, but also in selected patients with a normal sinus rhythm [26].

### 3.1. The Role of Atrial Dysfunction in Transthyretin Cardiac Amyloidosis

The prevalence of thromboembolic events and atrial fibrillation is higher in patients with transthyretin amyloidosis than in patients with light-chain amyloidosis. The higher incidence of atrial fibrillation in ATTR cardiac amyloidosis (CA) may be attributable to the restrictive atrial dysfunction caused by a slowly progressive direct infiltration of amyloid into the atrium, leading to the loss of atrial reservoir function and contractility [27]. Nochioka K et al. [28] investigated 124 patients with CA and a normal sinus rhythm (68 AL and 29 ATTRv, and 27 ATTRwt) using speckle tracking echocardiography—which directly measures the intrinsic myocardial deformation of the left atrium (LA), regardless of the loading conditions and the geometric hypotheses—and found an intrinsic myocardial deformation of the left atrium and an impairment of all phases of LA function in CA. Among the various subtypes, the peak LS and LA active emptying fraction was worse in ATTRwt compared to AL and ATTRv (*p* < 0.005). These findings were later corroborated by Versteylen MO et al. [27], who used conventional ultrasound and speckle tracking strain analysis in a population of 53 patients with cardiac amyloidosis and found a lower LA reservoir function in patients with transthyretin amyloidosis (7.4 (6.2%) vs. 13.6 (14.7%), *p* = 0.017), with no differences in LV systolic and diastolic pressures, atrial dimensions, and geometry between AL and ATTR subtypes. The authors also hypothesised a correlation between reduced LA function and cerebrovascular events independently of AF, though the number of thromboembolic events during follow-up was too small to allow further analysis.

**Table 1:** Thrombotic events in patients with ATTR amyloidosis.

Thrombotic Events	Study, Date	Population	Prevalence	Predisposing Factors
Intracardiac thrombi	Feng, 2007 [29]	55 ATTRwt 55 AL 4 AA	33%	-AL subtype (51% vs. 16%, *p* < 0.001) -AF -Left ventricular diastolic dysfunction
	Feng, 2009 [30]	56 ATTRwt 17 ATTRv 3 AA 80 AL	27%	-AL subtype (35% vs. 18%, *p* < 0.002) -AF -Lower left atrial appendage-emptying velocity -Diastolic dysfunction
	Martinez-Naharro, 2019 [31]	166 TTR 155 AL	6.2%	-AL subtype -AF -Atrial dilation -Higher ECV -Biventricular systolic dysfunction
	El-Am, 2019 [32]	25 ATTRwt 4 ATTRv	28%	-AF
Cerebrovascular events	Mitrani, 2020 [33]	290 TTR	6%	
	Donnellan, 2020 [34]	111 ATTRwt 271 ATTRv	16%	-Increased CHA_2_DS_2_VASC score -Non-anticoagulation
Cerebrovascular events, peripheral embolism	Cappelli, 2020 [35]	199 ATTRwt 73 ATTRv 134 AL	7.6%	-AF -Left ventricular ejection fraction < 50% -CHA_2_DS_2_VASC score > 2 -CKD
	Bukhari, 2021 [36]	77 ATTRwt 68 ATTRwt-AF	36.8% (ATTRwt-AF)	-AF
Intracardiac thrombi, cerebrovascular events, peripheral embolism	Vilches, 2022 [22]	1191 ATTR-CM	16.2%	-AF -Non-anticoagulation -Age -African-American race -Peripheral vascular disease

ATTRwt, wild-type transthyretin amyloidosis; ATTRv, hereditary transthyretin amyloidosis; AL, light-chain amyloidosis; TTR, transthyretin; AF, atrial fibrillation; CM, cardiomyopathy; ECV, extracellular volume; CKD, chronic kidney disease.

### 3.2. Intracardiac Thrombi 

Intracardiac thrombi are the most frequent thromboembolic event in CA, and their prevalence varies according to the screening approach. Feng D et al. [29] analysed a cohort of 116 autopsy or explant CA cases, of whom 55 had AL amyloidosis, 55 ATTRwt, 4 AA, and 2 ATTRv. Intracardiac thrombosis was found in 33% of cases. Analysing the different subpopulations, patients with AL amyloidosis, albeit younger and with a lower prevalence of AF, had significantly more intracardiac thrombi (51% vs. 16%; *p* < 0.001) and more fatal embolic events (26% vs. 8%; *p* < 0.03) than those with ATTR and AA. These findings were confirmed in a subsequent study by Feng D et al. [30], where, among 156 CA patients (*n* = 80 with AL, *n* = 76 with ATTR) undergoing transoesophageal echocardiography, 27% showed intracardiac thrombi. Patients with AL amyloidosis more frequently developed intracardiac thrombi than those with ATTR amyloidosis (35% vs. 18%; *p* = 0.02), despite being younger and with less frequent AF. From the multivariable analysis, the factors associated with an increased risk of intracardiac thrombosis were AF, poor left ventricular diastolic function, and lower left atrial appendage-emptying velocity. Martinez-Naharro A et al. [31] used cardiovascular magnetic resonance to assess the prevalence of intracardiac thrombi in 324 patients with CA: 166 had ATTR, 155 AL amyloidosis, 2 apolipoprotein AI amyloidosis, and 1 apolipoprotein A-IV amyloidosis. The prevalence of intracardiac thrombi was 6.2% in the overall population, 5.2% in AL, and 7.2% in ATTR (*p* = 0.45). The prevalence of thrombi in patients with AF/flutter was high: 13.1% overall, 9.1% in AL-CA, and 14.3% in ATTR-CA (*p* = 0.52), with AF being more frequent in ATTR-CA than AL-CA (46.4% vs. 14.2%, *p* < 0.001). Patients with a normal sinus rhythm also had intracardiac thrombi: 4.5% in AL-CA and 1.1% in ATTR-CA (*p* = 0.11). El-Am EA et al. [32] examined the outcomes of direct current cardioversion in patients with CA, including 29 (50%) with AL-CA, 25 (43%) with ATTRwt, and 4 (7%) with ATTRv. Patients with CA had a higher cardioversion cancellation rate (28% vs. 7%; *p* < 0.001) than the control patients due to intracardiac thrombi detected on the transoesophageal echocardiogram (13 of 16 (81%) vs. 2 of 8 (25%); *p* = 0.02). Interestingly, 4 of 13 of the CA patients (31%) with intracardiac thrombi received adequate anticoagulation for ≥3 weeks and 2 of 13 (15%) had an arrhythmia duration of <48 h.

### 3.3. Cerebrovascular Events

Unlike intracardiac thrombosis, data on arterial thromboembolic events in CA are scarce. Cerebrovascular events (e.g., transient ischaemic attach (TIA) and ischaemic stroke (IS)) are the most frequent after intracardiac thrombi. Mitrani LR et al. [33] analysed 290 patients with ATTR reporting thromboembolic events in 6%, IS in 9%, and TIA in 8%, all with AF. A retrospective cohort study by Donnellan E et al. [34], investigating the factors associated with AF development and outcome in a population of 382 patients with ATTR-CA (111 ATTRwt and 271 ATTRv), found that 63 (16%) patients had cerebrovascular events, of whom 53 (20%) had AF and 10 (9%) did not have AF and benefitted from a high CHA_2_DS_2_-VASc and the absence of anticoagulant therapy.

### 3.4. Cerebrovascular Events and Peripheral Embolism

Cappelli F et al. [35] investigated the occurrence of cerebrovascular events and peripheral embolism in 406 patients with CA: 134 AL-CA, 73 ATTRv-CA, and 199 ATTRwt-CA. A total of 31 patients (7.6%) had arterial thromboembolism: 29/31 with cerebrovascular events (21 IS and 8 TIA) and 1 mesenteric and 1 femoral embolism. Among these, 10 (32.2%) had a normal sinus rhythm and no history of AF. No significant differences in age, sex, and type of CA were found between patients with or without arterial thromboembolic events. Interestingly, anticoagulation therapy was not associated with a significantly reduced risk of thromboembolic events, whereas the only predictor of such events was a CHA_2_DS_2_-VASC score of ≥3. The study by Bukhari S et al. [36] confirmed an elevated thromboembolic risk in patients with ATTRwt-CA and AF by assessing the prevalence of AF, as well as the incidence and predictors of systemic thromboembolism (i.e., TIA, IS, or peripheral embolism). AF was present in 88% of patients with ATTRwt-CA, and the incidence of thromboembolism was higher in ATTRwt-CA patients with AF than in the controls (36.8% vs. 19.5%; *p* = 0.02; unadjusted hazard ratio 2.03 (95% CI, 1.10–3.85); *p* = 0.03), despite a lower CHA_2_DS_2_-VASc score. It emerged that ATTRwt-CA may therefore be considered a predictor of thromboembolism independent of the CHA_2_DS_2_-VASc score and left atrial volume index, potentially with important therapeutic implications.

### 3.5. Intracardiac Thrombi, Cerebrovascular Events, and Peripheral Embolism

Vilches S et al. [22] evaluated 1191 patients with transthyretin amyloid cardiomyopathy (ATTR-CM) and showed a progressively increasing risk of embolic events from 0 events per 100 patients-year in patients with a normal sinus rhythm and on anticoagulation therapy, to patients with a normal sinus rhythm not on anticoagulation therapy (1.3 events per 100 patients-year), to patients with AF and on anticoagulation therapy (1.7 events per 100 patients-year), up to 4.8 events per patients-year in those with AF not on anticoagulation. In this cohort of patients, the CHA_2_SD_2_-VASc score had a limited predictive value for embolic events in patients with ATTR-CA with and without AF, and vitamin K antagonists (VKAs) and direct oral anticoagulants (DOACs) appeared equally as effective in preventing embolism in this particular setting. Conversely, the rate of embolism was significantly higher in patients with labile INR or therapeutic time, in the range of <60%, who require adequate control of anticoagulant therapy. Further studies are needed to ascertain which individuals with ATTR-CA and without AF may benefit from specific screening or early anticoagulation treatment.

## 4. Bleeding Events in Transthyretin Amyloidosis 

### 4.1. Spontaneous Bleeding Manifestations: Case Reports

Bleeding manifestations are relatively frequent in AL amyloidosis [37]. However, several cases related to ATTR amyloidosis have been recently described, due to amyloid deposits in vessel walls causing the acceleration of microcalcification and vessel rupture. The most common dermatological findings are purpura and ecchymosis, caused by vascular fragility and wall damage possibly due to amyloid deposition and subsequent bleeding diathesis [38]. Some case reports appear to confirm these findings, such as the case of a 75-year-old man hospitalised for heart failure who presented with periorbital ecchymosis (raccoon eye) and shoulder swelling (pad sign) who was eventually diagnosed with ATTRwt-CA by cardiac biopsy [39]; and the case of a 69-year-old woman with periorbital purpura who, after echocardiography, was diagnosed with ATTRv-CA with the genetic variant Thr80Ala [40]. There have also been reports in the literature of gastrointestinal bleeding, obstruction, or perforation as a possible result of amyloid deposition in the gastrointestinal mucosa; a 79-year-old man with intra-abdominal haemorrhage underwent emergency partial resection of the transverse colon and the postoperative pathological examination of tissue samples led to the diagnosis of ATTR amyloidosis [41]. Urinary tract bleeding was reported in a patient with severe haematuria who was diagnosed with ATTRwt by cystoscopy and bladder biopsy. Amyloid can build up under the superficial mucosa or in the vasculature of the urinary tract, potentially causing massive bleeding [42]. Cases of retinal haemorrhage also have been reported in patients with ATTR amyloidosis due to vascular endothelial cell damage [43,44].

### 4.2. Bleeding While Undergoing DOACs vs. VKAs

Clotting abnormalities have been described mainly in AL amyloidosis, including prolonged prothrombin time, activated partial thromboplastin time, and FX deficiency; there are little data on ATTR amyloidosis [21]. The bleeding tendency in patients with ATTR amyloidosis is generally less severe than that in patients with AL and AA, though anticoagulation can exacerbate it. Therefore, the potential benefits of anticoagulation must be weighed carefully against potential bleeding complications [21,38]. A study by Mitrani RL et al. [33] found no differences in the combined outcome of IS, TIA, major bleeding, or death in patients with ATTR-CA and AF treated with warfarin vs. DOACs. However, labile INR was observed in 87% of patients and the incidence rate of major bleeding events was 3.7 per 100 person-years in this study vs. 2.2–3.9 in the general population. Cariou E et al. [45] analysed 273 patients with CA and a history of atrial arrhythmias on long-term anticoagulant therapy: 69 (25%) AL amyloidosis, 179 (66%) ATTRwt, and 25 (9%) ATTRv. Overall, 147 (54%) patients received VKA and 126 (46%) DOAC therapy. In the ATTRwt subgroup, the VKA-treated patients had a significantly higher bleeding risk than the DOAC-treated patients (14% vs. 2%, *p* < 0.001), likely due to more impaired renal function, whereas there were no differences in ischaemic events and no strokes occurred during follow-up. On the other hand, the bleeding risk in patients with AL amyloidosis was similar in the two groups (22% vs. 13% for VKAs and DOACs, respectively; *p* = 0.449), with no strokes during follow-up.

### 4.3. Type of Haemorrhagic Events According to Anticoagulant Therapy

The most frequent haemorrhagic events are haemorrhagic strokes and major extracranial bleeding [Table 2]. These events may occur in patients undergoing anticoagulation with no differences between direct oral anticoagulants and warfarin, as highlighted by Bukhari et al. [36], who reported haemorrhagic strokes in 4.4% and major extracranial bleeding in 7.3% of ATTRwt patients with AF. Major haemorrhagic events during anticoagulation were also reported by Vilches et al. [22], who studied 273 patients with AF receiving VKAs and 216 receiving DOACs during a median follow-up of 14.2 months. Overall, 32 haemorrhagic events occurred: 18 with VKAs and 14 with DOACs; major gastrointestinal and intracranial bleedings occurred in 46.9% and 12.5% of cases, respectively.

### 4.4. Amyloid Angiopathy Associated with an Increased Fragility of Blood Vessels

Microcalcifications are a common occurrence in cardiac ATTR amyloidosis (CA-ATTR) that are irregularly distributed and most pronounced near the fibrous body. Therefore, they are not strictly associated with amyloid deposits but are often found in collagenous areas. These clouds of microcalcifications explain the cardiac uptake of the bone tracers observed in CA-ATTR scintigraphy with technetium-99 [46]. Notably, histopathology examinations, CT, and MRI have shown vascular microcalcifications in hereditary CAA, though the underlying mechanisms are not yet fully understood [47]. These microcalcifications are most likely a secondary complication of CAA not directly caused by amyloid deposition and possibly induced by the extracellular osteopontin trapped in the fibrotic vessel wall. Vascular microcalcifications in amyloid-laden vessels may cause rupture, thus leading to both asymptomatic microbleeds and lobar ICH [48]. This mechanism is comparable to that observed in calcific aortic valve disease (CAVD), wherein the presence of apolipoprotein-related amyloid deposits near calcified areas suggests a possible interplay between the development of aortic valve stenosis, amyloid deposition, and calcification. These amyloid deposits may indeed contribute to enhance the inflammatory cycle in the aortic valve, including the remodelling of the extracellular matrix and the proliferation of myofibroblast- and osteoblast-like cells. Chemical changes in proteins in the local proinflammatory environment surrounding amyloid deposits could also play a role in calcification [49].

## 5. Conclusions

Both thrombotic and bleeding risks should be carefully stratified when dealing with patients with ATTR. Anticoagulation therapy is recommended in patients with AF regardless of the thrombotic risk. Nevertheless, there is little consensus among experts regarding the appropriate therapeutic management of patients with ATTR with a normal sinus rhythm or at risk of peripheral thromboembolic events [50,51]. Moreover, even though the bleeding risk in ATTR amyloidosis is generally less severe than in patients with AL, anticoagulation may exacerbate it. Therefore, the risk of bleeding must be thoroughly evaluated before initiating anticoagulant therapy given that there are currently no randomised studies that evaluate the optimal anticoagulant strategy (DOACs vs. VKAs) in transthyretin amyloidosis. In fact, the mechanisms underlying thrombotic and bleeding diatheses in ATTR amyloidosis are not yet fully understood. Further studies are needed to ascertain any coagulation abnormalities that may contribute to the development of thrombotic and bleeding events, and their impact on the decision to initiate anticoagulant therapy.

## Figures and Tables

**Table 2:** Bleeding events during anticoagulation.

Studies, Date	Population	Type of Bleeding	Prevalence	Comments
Feng, 2009 [30]	56 ATTRwt 17 ATTRv 3 AA 80 AL	Massive GI bleedings	1.9%	
Mitrani, 2020 [33]	290 ATTR	Miscellany	7%	Labile INR
Cariou, 2021 [45]	179 ATTRwt 25 ATTRv 69 AL	Miscellany	15% Minor bleedings 8%; Major bleedings 7%	VKAs vs. NOACs (14% vs. 2%)
Bukhari, 2021 [36]	77 ATTRwt	Miscellany	11.7% Haemorrhagic stroke 4.4% Major extracranial bleedings 7.3%	No difference between ATTRwt-AF and AF-control
Vilches, 2022 [22]	1191 ATTR-CM	Miscellany	6.5% GI bleedings 46.9% Intracranial bleedings 12.5%	No difference between ATTRwt-AF and AF-control

ATTRwt, wild-type transthyretin amyloidosis; ATTRv, hereditary transthyretin amyloidosis; AL, light-chain amyloidosis; TTR, transthyretin; CM, cardiomyopathy; GI, gastrointestinal bleeding; INR, international normalised ratio; VKAs, vitamin K antagonist; NOACs, new oral anticoagulants; AF, atrial fibrillation.

## Data Availability

Not applicable.
